# 继发性急性髓系白血病的治疗反应和结局及其影响因素分析

**DOI:** 10.3760/cma.j.issn.0253-2727.2023.02.007

**Published:** 2023-02

**Authors:** 玲 马, 婷 赵, 育红 陈, 浩 江, 兰平 许, 晓辉 张, 昱 王, 于谦 孙, 晓冬 莫, 晓军 黄, 倩 江

**Affiliations:** 北京大学人民医院、北京大学血液病研究所、国家血液系统疾病临床医学研究中心 100044 Peking University People's Hospital, Peking University Institute of Hematology, National Clinical Research Center for Hematologic Disease, Beijing 100044, China

**Keywords:** 白血病，髓系，急性, 继发性白血病, 早期治疗反应, 生存, 预后, Leukemia, myeloid, acute, Secondary leukemia, Early response, Survival, Prognosis

## Abstract

**目的:**

探讨成人继发性急性髓系白血病（sAML）的治疗反应和结局及其影响因素。

**方法:**

回顾性分析2008年1月至2021年2月北京大学人民医院收治的≤65岁sAML的连续病例，包括治疗相关AML（t-AML）、未明原因血细胞减少后AML、继发于骨髓增生异常综合征（MDS）的AML（post-MDS-AML）和继发于骨髓增殖性肿瘤（MPN）的AML（post-MPN-AML）的临床特征、治疗反应、复发和生存，采用二元Logistic模型分析治疗反应影响因素，Cox回归模型分析结局影响因素。

**结果:**

共纳入155例患者，t-AML、未明原因血细胞减少后AML、post-MDS-AML、post-MPN-AML组分别为38、46、57、14例。152例诱导化疗后可评估疗效患者中，首疗程诱导治疗后形态学无白血病状态（MLFS）率为47.4％，四组分别为57.9％、54.3％、40.0％、23.1％（*P*＝0.076）；最终MLFS率为63.8％，四组分别为73.3％、69.6％、58.2％、38.5％（*P*＝0.084）。多因素分析显示，全部患者中，男性（*OR*＝0.4，95％*CI* 0.2～0.9，*P*＝0.038；*OR*＝0.3，95％*CI* 0.1～0.8，*P*＝0.015）、SWOG非预后良好组（*OR*＝0.1，95％*CI* 0.1～0.6，*P*＝0.014；*OR*＝0.1，95％*CI* 0.1～0.3，*P*＝0.004）以及初始诱导弱化疗（*OR*＝0.1，95％*CI* 0.1～0.3，*P*＝0.003；*OR*＝0.1，95％*CI* 0.1～0.2，*P*＝0.001）是不利于首疗程诱导治疗获得完全缓解（CR）及最终获得CR的因素；此外，初诊时PLT<45×10^9^/L（*OR*＝0.4，95％*CI* 0.2～0.9，*P*＝0.038）、LDH≥258 U/L（*OR*＝0.3，95％*CI* 0.1～0.7，*P*＝0.005）亦为影响最终获得CR的独立危险因素。94例获得MLFS患者中，46例行异基因造血干细胞移植。中位随访18.6个月，持续化疗患者3年无复发生存（RFS）率和总生存（OS）率分别为25.4％、37.3％，移植患者3年RFS率和OS率分别为58.2％、64.3％。在获得MLFS患者中，多因素分析显示，年龄≥46岁（*HR*＝3.4，95％*CI* 1.6～7.2，*P*＝0.002；*HR*＝2.5，95％*CI* 1.1～6.0，*P*＝0.037）、初诊时外周血原始细胞≥17.5％（*HR*＝2.5，95％*CI* 1.2～4.9，*P*＝0.010；*HR*＝4.1，95％*CI* 1.7～9.7，*P*＝0.002）、单体核型（*HR*＝4.9，95％*CI* 1.2～19.9，*P*＝0.027；*HR*＝28.3，95％*CI* 4.2～189.5，*P*＝0.001）为影响RFS和OS的共同不利因素；此外，首疗程诱导治疗获得CR（*HR*＝0.4，95％*CI* 0.2～0.8，*P*＝0.015）及接受移植（*HR*＝0.4，95％*CI* 0.2～0.9，*P*＝0.028）与较长的RFS期显著相关。

**结论:**

与t-AML和未明原因血细胞减少后AML相比，post-MDS-AML和post-MPN-AML缓解率低、预后差。男性、初诊低PLT、高LDH、SWOG非预后良好组及初始诱导弱化疗是影响成年sAML获得缓解的不利因素，年龄≥46岁、外周血原始细胞比例高、单体核型是影响总体结局的不利因素，移植及诱导缓解达CR与更长的RFS期显著相关。

## Abstract (en)

**Objective:**

To evaluate treatment responses, outcomes, and prognostic factors in adults with secondary acute myeloid leukemia（sAML）.

**Methods:**

Between January 2008 and February 2021,date of consecutive cases of younger than 65 years of adults with sAML were assessed retrospectively. Clinical characteristics at diagnosis, treatment responses, recurrence, and survival were evaluated. Logistic regression and Cox proportional hazards model were employed to determine significant prognostic indicators for treatment response and survival.

**Results:**

155 patients were recruited, including 38，46，57，14 patients belonging to t-AML, and AML with unexplained cytopenia，post-MDS-AML, and post-MPN-AML，respectively. In the 152 evaluable patients, the rate of MLFS after the initial induction regimen was 47.4％, 57.9％, 54.3％, 40.0％, and 23.1％ in the four groups（*P*＝0.076）. The total rate of MLFS after the induction regimen was 63.8％, 73.3％, 69.6％, 58.2％, and 38.5％（*P*＝0.084）, respectively. Multivariate analysis demonstrated that male gender（*OR*＝0.4, 95％ *CI* 0.2–0.9, *P*＝0.038 and *OR*＝0.3, 95％ *CI* 0.1–0.8, *P*＝0.015）, SWOG cytogenetic classification into unfavorable or intermediate（*OR*＝0.1, 95％ *CI* 0.1–0.6, *P*＝0.014 and *OR*＝0.1, 95％ *CI* 0.1–0.3, *P*＝0.004）and receiving low-intensity regimen as induction regimen（*OR*＝0.1, 95％ *CI* 0.1–0.3, *P*＝0.003 and *OR*＝0.1, 95％*CI* 0.1–0.2, *P*＝0.001）were typical adverse factors impacting the first CR and the final CR; PLT<45 × 10^9^/L（*OR*＝0.4, 95％*CI* 0.2–0.9, *P*＝0.038）and LDH ≥258 U/L（*OR*＝0.3, 95％*CI* 0.1–0.7, *P*＝0.005）were independent factors for CR. Among the 94 patients with achieving MLFS, 46 cases had allogeneic hematopoietic stem cell transplantation. With a median follow-up period of 18.6 months, the probabilities of relapse-free survival（RFS）and overall survival（OS）at 3 years were 25.4％ and 37.3％ in patients with transplantation, and in patients with chemotherapy, the probabilities of RFS and OS at 3-year were 58.2％ and 64.3％, respectively. At the time of achieving MLFS, multivariate analysis revealed that age ≥46 years（*HR*＝3.4, 95％*CI* 1.6–7.2, *P*＝0.002 and *HR*＝2.5, 95％*CI* 1.1–6.0, *P*＝0.037）, peripheral blasts ≥17.5％ at diagnosis（*HR*＝2.5, 95％*CI* 1.2–4.9, *P*＝0.010 and *HR*＝4.1, 95％*CI* 1.7–9.7, *P*＝0.002）, monosomal karyotypes（*HR*＝4.9, 95％*CI* 1.2–19.9, *P*＝0.027 and *HR*＝28.3, 95％*CI* 4.2–189.5, *P*＝0.001）were typical adverse factors influencing RFS and OS. Furthermore, CR after induction chemotherapy（*HR*＝0.4, 95％*CI* 0.2–0.8, *P*＝0.015）and transplantation（*HR*＝0.4, 95％*CI* 0.2–0.9, *P*＝0.028）were substantially linked to longer RFS.

**Conclusion:**

Post-MDS-AML and post-MPN-AML had lower response rates and poorer prognoses than t-AML and AML with unexplained cytopenia. In adults with male gender, low platelet count, high LDH, and SWOG cytogenetic classification into unfavorable or intermediate at diagnosis, and receiving low-intensity regimen as the induction regimen predicted a low response rate. Age ≥46 years, a higher proportion of peripheral blasts and monosomal karyotype had a negative effect on the overall outcome. Transplantation and CR after induction chemotherapy were greatly linked to longer RFS.

业内已证实，与初诊急性髓系白血病相比，肿瘤放化疗后发生的治疗相关急性髓系白血病（t-AML）和继发于先前血液系统克隆性疾病的急性髓系白血病（AHD-AML）对标准化疗缓解率低，复发率高，预后差，且多数被排除出临床试验[1]。此类疾病多为老年患者，异质性较大，临床特点及预后影响因素国外多有报道，但国内报道较少。曾经存在一系或两系血细胞减少但未明确诊断、后期发生AML的研究也少有报道。为此，我们回顾性分析t-AML、继发于骨髓增生异常综合征（MDS）的AML（post-MDS-AML）、继发于骨髓增殖性肿瘤（MPN）的AML（post-MPN-AML）以及既往具有未明原因血细胞减少但诊断未明的AML的临床特征、治疗反应及预后，探讨相关影响因素。

## 病例与方法

一、病例

回顾性收集2008年1月至2021年2月于北京大学人民医院确诊并接受治疗，≤65岁的治疗相关AML、post-MDS-AML、post-MPN-AML以及未明原因血细胞减少后AML连续病例。在本研究中，未明原因血细胞减少定义为既往血细胞一系或多系减少（包括WBC、HGB、PLT）超过3个月，除外缺铁性贫血、巨幼细胞性贫血、再生障碍性贫血等血液系统疾病，除外药物或自身免疫性疾病相关的血细胞减少等疾病，未能明确诊断的疾病状态。所有患者均通过既往病史及骨髓MICM（细胞形态学、免疫学、遗传学及分子生物学）确诊，符合2016版WHO诊断标准[2]。

二、实验室检测

1. 细胞遗传学分析：骨髓标本经G显带法分析染色体核型，根据《人类细胞遗传学国际命名体制（ISCN，1995）》进行核型描述。单体核型定义为在一个克隆内存在2个常染色体单体或1个常染色体单体伴有其他常染色体结构异常。参考美国西南肿瘤协作组（SWOG）标准[3]进行危险度预后分组。

2. 免疫学分析：采用8色免疫标记法流式细胞术（FCM）检测骨髓细胞免疫表型。CD34-FITC/CD13-PE/CD117-PerCP/CD33-APC/HLA-DR-APC-CY7/CD45-V500，CD38/CD7/CD56抗体用于初步诊断为AML的患者[4]。

3. 分子生物学检测：分子生物学检测包括AML1-ETO、CBFβ-MYH11、PML-RARα、NPM1、FLT3-ITD基因突变、混合系白血病（MLL）相关融合基因（包括MLL-AF4、MLL-AF6、MLL-AF9、MLL-AF10、MLL-ELL、MLL-ENL、MLL-AF1p、MLL-AF1q、MLL-SEPT9）和WT1 mRNA。检测参照本所常规方法[5]。

三、治疗方案

1. 诱导治疗：诱导治疗方案包括IA方案（去甲氧柔红霉素10 mg/m^2^第1～3天，阿糖胞苷100 mg/m^2^第1～7天）、HAA方案（高三尖杉酯碱2 mg/m^2^第1～7天，阿克拉霉素20 mg/d第1～7天，阿糖胞苷100 mg/m^2^第1～7天）、CAG方案（阿糖胞苷12 mg/m^2^第1～14天，阿克拉霉素20 mg/d第1～4天或10 mg/d第1～8天，G-CSF 300 µg/d第1～14天）及弱化疗，弱化疗包括AA方案（阿克拉霉素20 mg/d第1～7天，阿糖胞苷100 mg/m^2^第1～7天）、MA方案（米托蒽醌2 mg/d第1～7天或4 mg/d第1～5天，阿糖胞苷100 mg/m^2^第1～7天）、小剂量HA方案（高三尖杉酯碱1 mg/d第1～14天，阿糖胞苷12 mg/m^2^第1～14天）、半量CAG方案（阿糖胞苷12 mg/m^2^第1～7天，阿克拉霉素10 mg/d第1～4天，G-CSF 300 µg第1～7天）、去甲基化药物单药（如阿扎胞苷75 mg/m^2^第1～7天或地西他滨20 mg/m^2^第1～5天）至少2个疗程。

2. 缓解后治疗：包括含中高剂量阿糖胞苷（1～2 g/m^2^，每12 h 1次，第1～3天）的方案、原方案巩固或MA、HAA、CAG等方案。根据患者有无供者、自身状况、社会经济因素及个人家庭意愿，在获得缓解、巩固1～2个疗程后进行单倍体异基因造血干细胞移植。

四、疗效评估指标

每次化疗结束后4周左右进行骨髓穿刺检查。

1. 治疗反应：根据2017年欧洲白血病网（ELN）指南[6]定义。①形态学无白血病状态（MLFS）：骨髓原始细胞<5％（至少计数200个细胞），无伴Auer小体的原始细胞或无髓外白血病持续存在。②完全缓解（CR）：满足MLFS，且中性粒细胞绝对计数≥1.0×10^9^/L，PLT≥100×10^9^/L。③完全缓解伴血细胞计数未完全恢复（CRi）：满足MLFS，但仍有中性粒细胞减少（<1.0×10^9^/L）或血小板减少（<100×10^9^/L）。④部分缓解（PR）：血细胞计数符合CR标准，骨髓原始细胞比例5％～25％（同时应较治疗前下降50％以上）。⑤客观缓解率（ORR）：CR率+CRi率+PR率。

2. 结局:复发定义为CR或CRi患者外周血中又出现白血病细胞，或骨髓中原始细胞≥5％或出现新的病态造血，或髓外出现形态学可证实的白血病细胞。无复发生存（RFS）期仅用于评价达到MLFS的患者，定义为自达到MLFS至患者复发、因各种原因死亡或末次随访的时间。总生存（OS）期定义为自AML诊断之日至患者因各种原因死亡或末次随访日的时间。

五、随访

主要采用定期门诊及电话随访方式进行，随访截止时间为2021年4月。

六、统计学处理

统计采用SPSS 26.0软件进行。采用描述性统计，分析患者人口学和临床特征。样本组间比较，分类变量进行卡方检验，连续变量进行Kruskal-Wallis非参数检验。其中，连续变量采用中位数做二分类变量进行分析。对治疗反应的影响因素，使用二元Logistic模型进行单因素分析，将*P*<0.2的因素纳入Logistic回归模型进行多因素分析。影响RFS和OS的因素采用Kaplan-Meier生存分析法进行Log-rank检验，将*P*<0.2的因素代入Cox回归模型进行多因素分析。*P*<0.05为差异有统计学意义。

## 结果

一、患者特征

共收集2008年至2021年诊治的155例连续患者，其中t-AML38例，未明原因血细胞减少后AML 46例，post-MDS-AML 57例，post-MPN-AML 14例，患者临床特征见表1。四组间性别、诊断时WBC、HGB、LDH、骨髓原始细胞比例、SWOG预后分层差异均有统计学意义（*P*值均<0.05）。

**表1:** 继发性急性髓系白血病（AML）患者一般临床特征比较

指标	t-AML（38例）	未明原因血细胞减少后AML（46例）	post-MDS-AML（57例）	post-MPN-AML（14例）	*P*值
男性［例（％）］	14（36.8）	22（47.8）	35（61.4）	10（71.4）	0.047
年龄［岁，*M*（范围）］	47（17～64）	46（22～62）	49（19～65）	49（31～62）	0.696
WBC［×10^9^/L，*M*（范围）］	5.9（1.4～113.8）	5.4（0.7～90.1）	2.45（0.7～51.6）	16.0（4.2～89.4）	<0.001
HGB［g/L，*M*（范围）］	85（39～130）	85.5（42～141）	73（30～139）	70（34～150）	0.034
PLT［×10^9^/L，*M*（范围）］	45（4～437）	42.5（9～291）	44（1～372）	70.5（10～372）	0.232
LDH［U/L，*M*（范围）］	254.5（139～3 539）	303（135～4 185）	226（103～3 902）	473.5（93～2 539）	0.043
ALB［g/L，*M*（范围）］	38.15（31.8～48.8）	41.1（26.0～50.3）	40.4（29.6～47.9）	40.65（27.0～49.3）	0.082
骨髓原始细胞［％，*M*（范围）］	50.5（21～96）	41（10～99）	27（3～97）	26（9～64）	<0.001
外周血原始细胞［％，*M*（范围）］	22.5（0～96）	13（0～94）	7（0～92）	11（0～88）	0.107
WT1水平［％,*M*（范围）］	20.4（0～148.4）	17.8（0～137.1）	17.7（0～246.3）	5.6（0～64.4）	0.292
SWOG分组［例（％）］					0.004
低危	8（21.1）	4（8.7）	1（1.8）	0（0）	
中危	15（39.5）	31（67.4）	36（63.2）	5（35.7）	
高危	11（28.9）	6（13.0）	14（24.6）	4（28.6）	
未知	4（10.5）	5（10.9）	6（10.5）	5（35.7）	
单体核型［例（％）］	4（11.1）	1（2.4）	5（9.1）	1（7.7）	0.424
基因突变［例（％）］					
NPM1阳性	4（10.5）	7（15.9）	5（9.4）	2（16.7）	0.744
FLT3-ITD阳性	5（13.2）	3（6.8）	8（15.1）	2（18.2）	0.545
MLL融合阳性	9（24.3）	6（14.3）	4（7.5）	2（16.7）	0.174

**注** t-AML：治疗相关AML；post-MDS-AML：继发于骨髓增生异常综合征的AML；post-MPN-AML：继发于骨髓增殖性肿瘤的AML；LDH：乳酸脱氢酶；ALB：白蛋白

二、治疗反应及其影响因素

1. 缓解率：155例患者中，除2例直接移植外，153例接受诱导化疗，其中1例早期死亡。可评估治疗反应的152例患者中，首疗程诱导治疗后，ORR 53.9％（82/152），t-AML、未明原因血细胞减少后AML、post-MDS-AML、post-MPN-AML组ORR分别为63.2％（24/38）、60.9％（28/46）、47.3％（26/55）和30.8％（4/13）（*P*＝0.112）。72例（47.4％）获得MLFS，四组MLFS率分别为57.9％、54.3％、40.0％、23.1％（*P*＝0.076）。56例（36.8％）获得CR，四组CR率分别为50.0％、45.7％、25.5％、15.4％（*P*＝0.190）。

经历中位1（1～4）个疗程诱导化疗，最终ORR 64.5％（98/152），t-AML、未明原因血细胞减少后AML、post-MDS-AML、post-MPN-AML组ORR分别为73.7％（28/38）、71.7％（33/46）、58.2％（32/55）、38.5％（5/13）（*P*＝0.064）。97例（63.8％）获得MLFS，四组MLFS率分别为73.3％、69.6％、58.2％、38.5％（*P*＝0.084）。69例（45.4％）获得CR，四组CR率分别为57.9％、56.5％、32.7％、23.1％（*P*＝0.012）。

分析首疗程不同诱导方案的缓解率，采用IA、HAA、CAG、弱化疗方案组ORR分别为59.0％（23/39）、72.0％（18/25）、54.0％（34/63）、28.0％（7/25）（*P*＝0.015）；MLFS率分别为56.4％、64.0％、47.6％、16.0％（*P*＝0.003）；CR率分别为51.3％、56.0％、33.3％、4.0％（*P*<0.001）。

2. 识别治疗反应的影响因素：分析患者发病时特征［包括年龄、性别、疾病类型、WBC、HGB、PLT、乳酸脱氢酶（LDH）、白蛋白（ALB）、外周血和骨髓原始细胞比例、SWOG分组、是否有单体核型、初诊FLT3-ITD突变和NPM1突变状态］和初始诱导方案对MLFS和CR的影响。多因素分析显示，SWOG非预后良好组和初始诱导弱化疗是不利于首疗程诱导治疗获得MLFS的因素；初诊时LDH≥258 U/L和post-MDS-AML和post-MPN-AML是不利于最终获得MLFS的因素（表2）。

**表2:** 影响继发性急性髓系白血病（AML）患者诱导治疗后获得早期治疗反应的多因素分析

变量	首疗程诱导治疗后						最终诱导治疗后					
	MLFS			CR			MLFS			CR		
	*OR*	95% *CI*	*P*值	*OR*	95% *CI*	*P*值	*OR*	95% *CI*	*P*值	*OR*	95% *CI*	*P*值
性别（男/女）				0.4	0.2～0.9	0.038				0.3	0.1～0.8	0.015
PLT（<45×10^9^/L/≥45×10^9^/L）										0.4	0.2～0.9	0.038
LDH（≥258 U/L/<258 U/L）				0.4	0.2～1.0	0.060	0.4	0.2～0.9	0.035	0.3	0.1～0.7	0.005
SWOG分组（非预后良好/预后良好）	0.1	0.1～0.3	0.005	0.1	0.1～0.6	0.014				0.1	0.1～0.3	0.004
疾病分组									0.081			
t-AML（参考组）												
未明原因血细胞减少后AML							0.8	0.3～2.1	0.595			
post-MDS-AML							0.3	0.1～0.9	0.044			
post-MPN-AML							0.2	0.1～1.1	0.059			
诱导治疗方案			0.018			0.009			0.089			0.007
IA±HMA（参考组）												-
HAA±HMA	1.2	0.4～3.7	0.702	1.1	0.1～3.7	0.866	1.7	0.5～5.9	0.399	1.1	0.3～3.7	0.955
CAG±HMA	0.8	0.4～1.9	0.626	0.5	0.2～1.3	0.150	1.4	0.5～3.8	0.551	0.5	0.2～1.4	0.202
弱化疗	0.1	0.1～0.5	0.003	0.1	0.1～0.3	0.003	0.4	0.1～1.1	0.082	0.1	0.1～0.2	0.001

**注** MLFS：形态学无白血病状态；CR：完全缓解；LDH：乳酸脱氢酶；t-AML：治疗相关AML；post-MDS-AML：继发于骨髓增生异常综合征的AML；post-MPN-AML：继发于骨髓增殖性肿瘤的AML；IA：去甲氧柔红霉素+阿糖胞苷；HAA：高三尖杉酯碱+阿克拉霉素+阿糖胞苷；CAG：阿克拉霉素+阿糖胞苷+G-CSF；HMA：去甲基化药物

男性、SWOG非预后良好组和初始诱导弱化疗是不利于首疗程诱导治疗获得CR的因素；初诊时PLT<45×10^9^/L、LDH≥258 U/L、男性、SWOG非预后良好组和初始诱导弱化疗是不利于最终获得CR的因素。

三、结局及其影响因素

1. 复发与生存：152例患者，中位随访26.5（1.4～92.5）个月，存活者中位随访18.0（2.6～92.5）个月。55例未缓解者中位生存5.8（1.4～21.0）个月。94例获得MLFS的患者持续治疗，48例持续化疗患者中38例（79.2％）进行了中高剂量阿糖胞苷方案巩固化疗。获得MLFS的患者中t-AML、未明原因血细胞减少后AML、post-MDS-AML、post-MPN-AML组3年RFS率分别为31.9％、46.8％、36.9％、40.0％（*P*＝0.177，图1A），3年OS率分别为59.8％、47.4％、43.4％、50.0％（*P*＝0.976，图1B），差异均无统计学意义。随访期内，持续化疗患者中28例（58.3％）复发，22例（45.8％）死亡，其中20例死于复发，2例非复发死亡者均死于感染。46例获得MLFS患者在中位5.5个月时接受移植，8例（17.4％）在移植后中位5.6个月时复发，12例（26.2％）死亡，其中4例死于复发，8例死于移植相关并发症。持续化疗患者中位RFS期为14.5个月，中位OS期为26.7个月，接受移植患者中位RFS和OS期未达到。两组患者3年RFS率分别为25.4％和58.2％（*P*＝0.001），3年OS率分别为37.3％和64.3％（*P*＝0.024）（图2）。

**图1:**
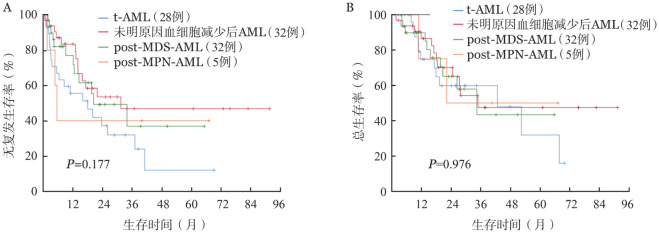
获得形态学无白血病状态及以上继发性急性髓系白血病（AML）患者的无复发生存（A）和总生存（B）曲线 **注** t-AML：治疗相关AML；post-MDS-AML：继发于骨髓增生异常综合征的AML；post-MPN-AML：继发于骨髓增殖性肿瘤的AML

**图2:**
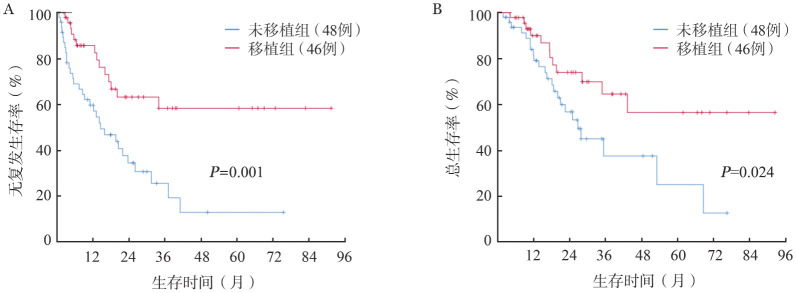
获得形态学无白血病状态及以上的继发性急性髓系白血病患者中移植组与未移植组无复发生存（A）和总生存（B）曲线

2. 识别影响复发与生存的因素：在获得MLFS的人群中，分析患者发病时特征、首疗程诱导治疗后缓解程度、巩固是否采用含中高剂量阿糖胞苷方案、缓解后是否行移植对复发和生存的影响。多因素分析显示，年龄≥46岁、初诊时外周血原始细胞≥17.5％、单体核型与较低的RFS率和OS率均显著相关；而初始诱导获得CR及接受移植与较长的RFS时间显著相关；巩固治疗含中高剂量阿糖胞苷有获得较长OS时间趋势（表3）。

**表3:** 影响继发性急性髓系白血病（AML）患者复发与生存的多因素分析

变量	RFS			OS		
	*HR*	95％ *CI*	*P*值	*HR*	95％ *CI*	*P*值
年龄（≥46岁/<46岁）	3.4	1.6～7.2	0.002	2.5	1.1～6.0	0.037
外周血原始细胞（≥17.5％/<17.5％）	2.5	1.2～4.9	0.010	4.1	1.7～9.7	0.002
伴单体核型（是/否）	4.9	1.2～19.9	0.027	28.3	4.2～189.5	0.001
完全缓解（是/否）	0.4	0.2～0.8	0.015			
巩固治疗含高剂量阿糖胞苷（是/否）				0.5	0.2～1.1	0.088
移植（是/否）	0.4	0.2～0.9	0.028			

**注** 在获得形态学无白血病状态及以上的人群中分析；RFS：无复发生存；OS：总生存

## 讨论

本研究通过回顾性数据分析发现与t-AML和未明原因血细胞减少后AML相比，post-MDS-AML和post-MPN-AML缓解率更低、预后更差。男性、初诊时高LDH、SWOG非预后良好组及初始诱导弱化疗是影响成人sAML患者获得缓解的不利因素，年龄≥46岁、外周血原始细胞比例高、单体核型是影响总体结局的不利因素，移植可以改善RFS，且诱导缓解深度亦与更长的RFS时间显著相关。

本研究显示未明原因血细胞减少后AML的临床特点不完全与post-MDS-AML相同，多介于t-AML与post-MDS-AML之间。t-AML女性更多见，这与女性更容易患妇科肿瘤相关。在SWOG分组方面，与既往本中心报道[9]的1 097例65岁以下的初治AML患者（16.8％）相比，t-AML、post-MDS-AML、post-MPN-AML显示出更高的SWOG高危患者比例（分别为28.9％、24.6％、28.6％），但三者之间差异无统计学意义；未明原因血细胞减少后AML中SWOG高危的比例较低（13.0％）。丹麦Granfeldt等[10]报道的1 567例接受强化化疗的AML患者中，t-AML 102例，其中58％为女性患者，同样高于初诊AML（45％女性），在SWOG分组中，post-MDS-AML、非MDS转化的sAML、t-AML中SWOG预后高危组的比例也高于初诊AML（25.4％、27％、39.8％和18.5％），但与本研究不同的是t-AML较其他sAML显示出更高比例的SWOG高危患者。

在国外的多个研究[10]–[14]中发现，相较初治AML患者，sAML患者诱导化疗后CR率明显减低，且在治疗模式相同情况下，t-AML较AHD-AML早期治疗反应更好，且不论年龄及染色体危险度，AHD-AML与治疗结局差及预后不良相关。国内卢绮思等[15]报告了35例治疗相关的血液肿瘤，其中t-AML 20例，同样发现治疗相关血液肿瘤患者预后极差。本研究数据同样显示t-AML获得MLFS的比例（73.7％）最高，post-MDS-AML次之（58.2％），post-MPN-AML最差（38.5％）。且既往存在血细胞减少病史的患者继发AML同样有较差的缓解率（71.7％），但较post-MDS-AML稍高。t-AML与未明原因血细胞减少后AML在首疗程诱导治疗后ORR类似（63.2％对60.9％），较post-MDS-AML（47.3％）、post-MPN-AML（30.8％）更高。与国外研究类似，t-AML在缓解率上优于前驱血液病病史的AML。而且在接受强化诱导治疗的sAML中，与本中心既往报道[16]的初治AML在1个疗程诱导治疗后66.2％的患者获得MLFS及以上疗效相比，sAML四组MLFS均明显下降，分别为57.9％、54.3％、40.0％、23.1％。同丹麦研究[10]类似。本中心数据支持在sAML诱导治疗中强化疗（IA/HAA/CAG）疗效优于弱化疗，且多因素分析发现诱导方案强弱是影响初始诱导治疗是否可获得缓解及诱导缓解深度的独立危险因素，提示缓解更依赖于强化疗。

既往研究显示sAML预后差，中位OS期在6个月到24个月不等[17]–[24]。2016年我们中心[25]配对分析了获得CR的t-AML，结果显示在CR_1_状态下进行移植的t-AML和初发AML患者3年OS率（66％、79％）、3年无白血病生存（LFS，64％、77％）率均明显提高，且3年非复发死亡（NRM）率差异无统计学意义（13％对9％），提示移植可改善t-AML患者的生存。本研究显示能获得缓解的sAML患者中位OS期为19.6个月，异基因造血干细胞移植明显优于单纯化疗，3年RFS率分别为58.2％和25.4％（*P*＝0.001），3年OS率分别为64.3％和37.3％（*P*＝0.024）。而且在多因素分析中，异基因造血干细胞移植是RFS的独立影响因素。

在AML96研究[26]中，分析了305例sAML，多因素分析发现年龄>60岁、PLT<50×10^9^/L、染色体核型高危、初诊NPM1突变阴性为影响OS、EFS的独立危险因素。本研究采用多因素分析，发现即使在sAML患者中，SWOG分组是否是预后良好组仍然可以影响诱导后缓解率及缓解深度，男性、初始LDH≥258 U/L以及初始诱导弱化疗为影响首疗程诱导获得CR及最终获得CR的独立危险因素。而年龄≥46岁、初诊外周血原始细胞比例高、单体核型为影响RFS和OS的独立危险因素，异基因造血干细胞移植为sAML患者带来RFS方面优势，且巩固治疗方案中含中高剂量阿糖胞苷可提高患者的OS率。

本研究有以下局限性：为回顾性研究，病例跨越年度大、药物可及性的限制以及化疗方案参差不齐，混杂因素影响大，本研究通过多因素分析尽量减小混杂因素影响。另外本研究ECOG评分缺失，四组患者样本量小，且大部分缺乏基因组学检测结果，主要从病史上分析，而和基因突变类型的相关性需要进一步深入研究。

总之，sAML缓解率低，预后差，SWOG预后良好组、强化诱导治疗缓解率高。年龄≥46岁、外周血原始细胞比例高及单体核型是影响患者总体预后的不利因素。强化诱导治疗、中高剂量阿糖胞苷巩固治疗、异基因造血干细胞移植可带来生存获益，65岁以下年轻成人sAML应强调标准剂量的规范诊治，不能一味减低治疗强度。
